# Metabolic potential for benzoate utilization revealed by the complete genome of *Sphingobacterium* sp. PAMC25397

**DOI:** 10.1128/mra.00242-26

**Published:** 2026-09-03

**Authors:** Min-Su Kim, Byeollee Kim, So-Ra Han, Hyun Lee, Hye Jin Jung, Jun Hyuck Lee, Tae-Jin Oh

**Affiliations:** 1 Department of Life Science and Biochemical Engineering, Graduate School SunMoon University 35022 , Asan, South Korea; 2 Genome-based BioIT Convergence Institute, Asan, South Korea; 3 Division of Computer Science and Engineering, SunMoon University 35022, Asan, South Korea; 4 Department of Pharmaceutical Engineering and Biotechnology, SunMoon University 35022, Asan, South Korea; 5 Division of Life Sciences, Korea Polar Research Institute 123591, Incheon, South Korea; Indiana University Bloomington, Bloomington, Indiana, USA

**Keywords:** *Sphingobacterium *sp., complete genome, benzoate degradation, Arctic bacteria

## Abstract

Here, we report the complete genome sequence of *Sphingobacterium* sp. PAMC25397, isolated from Ny-Ålesund, Spitsbergen, Norway. The genome is 5.2 Mb in length with a GC content of 37%. This genome harbors biodegradation-related genes involved in benzoate degradation.

## ANNOUNCEMENT

Aromatic compounds account for over 65% of mass-produced chemicals and organic pollutants (1). Benzoic acid is a common pollutant in dye effluents and food-processing wastewater (2). Microbial degradation of aromatics is important for waste treatment and bioremediation (3).

*Sphingobacterium* sp. PAMC25397 was isolated from animal excrement-associated debris collected in Ny-Ålesund, Spitsbergen, Norway (78.91502°N, 11.87143°E). Approximately 1 g of the sample was aseptically collected, cultured on pork-supplemented 0.1× nutrient agar at 4°C for 6 months, and purified by repeated streaking. The same single colony was used for both 16S rRNA gene amplification and whole-genome sequencing, and genomic DNA was extracted from R2A-grown cells using the DNeasy Blood & Tissue Kit (Qiagen, the Netherlands). The 16S rRNA gene was amplified using the universal primers 27F and 1492R and analyzed by Sanger sequencing (Cosmo Genetech, Korea). Taxonomic identification was based on comparison of the 16S rRNA sequence with the EzBioCloud database (4), and the closest hit was *Sphingobacterium* sp. ML3W (CP009278) (99.86%).

The same genomic DNA extract from PAMC25397 was used to prepare Illumina and Oxford Nanopore Technology (ONT) libraries for sequencing (Phyzen, Korea). Illumina libraries were prepared using the TruSeq DNA Nano Kit (Illumina, USA) and sequenced on a NovaSeq 6000 platform (2 × 150 bp paired-end reads). ONT libraries were prepared using the 1D Ligation Sequencing Kit (SQK-LSK110) and Native Barcoding Expansion Kit (EXP-NBD104) without size selection, sequenced on the GridION platform (FLO-MIN106, R9.4 flow cell), and basecalled with MinKNOW v22.12.7 and Guppy 6.4.6, both in high-accuracy mode. Pre-sequencing library QC was performed using Qubit and gel electrophoresis for ONT and qPCR and an Agilent 2100 Bioanalyzer (DNA 1000 chip) for Illumina. Illumina and Nanopore reads were processed using fastp v0.20.1 (default settings) and Filtlong v0.2.1 (–min_length 1000 and –keep_percent 95), respectively. ONT reads used for genome assembly comprised 53,309 reads, totaling 818.3 Mbp, with an N50 of 21,063 bp and approximately 159× coverage; Illumina reads comprised 8,644,901 reads, totaling 1,305.3 Mbp (Table 1).

**TABLE 1:** Raw sequence information

Parameters	Genomic features
ONT statistics	
Reads	53,309
Total	818.3 Mbp
N50	21,063 bp
Coverage	159×
Illumina statistics	
Reads	8,644,901
Total	1,305.3 Mbp

Following the Trycycler workflow (5), 12 subsampled data sets were independently assembled using Flye v2.9.2 (6), reconciled using Trycycler v0.5.4 (7) to generate a consensus assembly, and then polished with Medaka v1.12.1 (8), Polypolish v0.6.0 (9), and POLCA v0.3.1 (10). Circularization was verified using Trycycler (11), which refines the contig boundaries. Genome quality was assessed using QUAST v5.2.0 (12) and CheckM v1.2.2 (13). CheckM estimated the genome to be 100% complete, with 1.43% contamination. Taxonomic classification was determined using the ANI Calculator (14). Genome annotation was performed using Prokka v1.14.6 (15) and eggnog-mapper v2.1.12 (16). KEGG pathway reconstruction was performed using KEGG Mapper (reconstruction module) (17). All software parameters were set to default unless otherwise specified.

**Fig 1:**
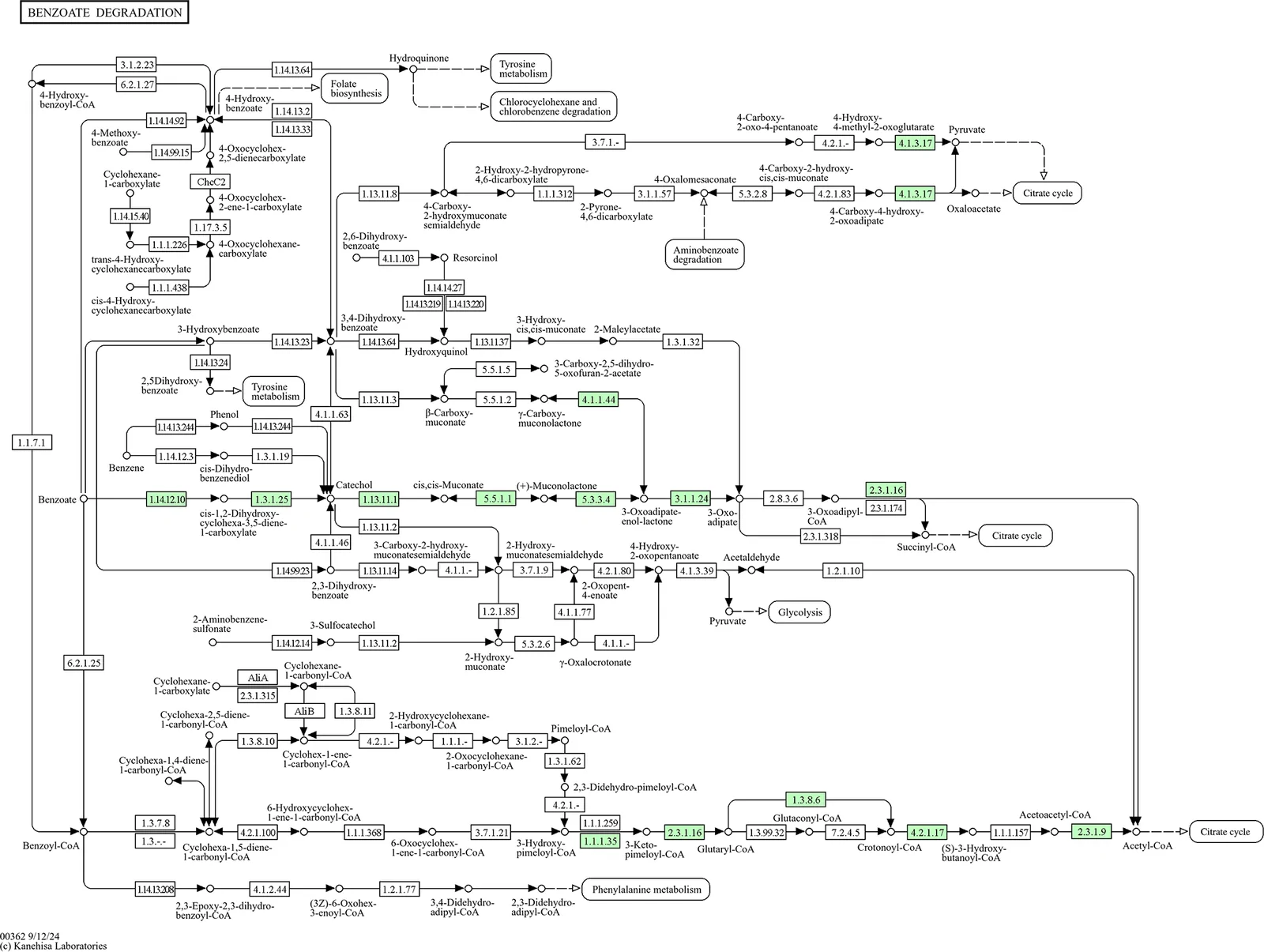
KEGG pathway reconstruction of benzoate degradation in *Sphingobacterium* sp. PAMC25397. Detected enzymes are highlighted in green.

The complete genome of PAMC25397 was assembled into a single circular contig of 5,157,497 bp and a GC content of 37%. ANI analysis revealed the highest similarity of 98.25% to *Sphingobacterium* sp. ML3W (GCA_000747525.1). Genome annotation identified a total of 4,387 protein-coding sequences, 89 tRNAs, and 1 transfer-messenger RNA (tmRNA. KEGG pathway reconstruction revealed multiple genes involved in benzoate degradation, suggesting genetic potential for this pathway (Fig. 1).

## Data Availability

The complete genome sequencing for *Sphingobacterium* sp. PAMC25397 has been deposited in GenBank under the GCA_983196555.1. The raw sequencing reads for *Sphingobacterium* sp. PAMC25397 have been deposited in the ENA under the project accession number PRJEB106065, sample accession number SAMEA121008656, experiment accession number ERX15535198, and run accession number ERR16145640. The genome annotation files for PAMC25397, including Prokka output files and eggnog-mapper functional annotation files, have been deposited in Figshare and are available at https://doi.org/10.6084/m9.figshare.32165154.
